# Exercise Training in Transgenic Mice Is Associated with Attenuation of Early Breast Cancer Growth in a Dose-Dependent Manner

**DOI:** 10.1371/journal.pone.0080123

**Published:** 2013-11-27

**Authors:** Jorming Goh, Jesse Tsai, Theo K. Bammler, Frederico M. Farin, Emma Endicott, Warren C. Ladiges

**Affiliations:** 1 Interdisciplinary Program in Nutritional Sciences, University of Washington, Seattle, Washington, United States of America; 2 Department of Comparative Medicine, University of Washington, Seattle, Washington, United States of America; 3 Department of Environmental and Occupational Health Sciences, University of Washington, Seattle, Washington, United States of America; Universidad Europea de Madrid, Spain

## Abstract

Epidemiological research suggests that regular physical activity confers beneficial effects that mediate an anti-tumor response and may reduce cancer recurrence. It is unclear what amount of physical activity is necessary to exert such a protective effect and what mechanisms are involved. We investigated the effects of voluntary wheel running on tumor progression and cytokine gene expression in the transgenic polyoma middle T oncoprotein (PyMT) mouse model of invasive breast cancer. Runners showed significantly reduced tumor sizes compared with non-runners after 3 weeks of running (p≤0.01), and the greater the running distance the smaller the tumor size (Pearson's r = −0.61, p≤0.04, R^2^ = 0.38). Mice running greater than 150 km per week had a significantly attenuated tumor size compared with non-runners (p≤0.05). Adipose tissue mass was inversely correlated with tumor size in runners (Pearson's r = −0.77, p = 0.014) but not non-runners. Gene expression of CCL22, a cytokine associated with recruitment of immunosuppressive T regulatory cells, was decreased in tumors of runners compared to non-runners (p≤0.005). No differences in tumor burden or metastatic burden were observed between runners and non-runners after ten weeks of running when the study was completed. We conclude that voluntary wheel running in PyMT mice correlates with an attenuation in tumor progression early during the course of invasive breast cancer. This effect is absent in the later stages of overwhelming tumor burden even though cytokine signaling for immunosuppressive regulatory T cells was down regulated. These observations suggest that the initiation of moderate exercise training for adjunctive therapeutic benefit early in the course of invasive breast cancer should be considered for further investigation.

## Introduction

Epidemiological studies have revealed a protective effect of physical activity on breast cancer-related mortality in women diagnosed with breast cancer [1], [2], whereby increased physical activity was associated with reduced recurrence and mortality [3]. These observations consistently suggest a potential dose-dependent effect of physical activity on breast cancer outcomes. In studies involving breast cancer survivors, physical activity patterns are measured by memory recall of their latest and life-time bouts of physical activity, which is subjective and prone to recall bias. Therefore, interpretation of physical activity measurements from epidemiological studies, while meaningful, has causal limitations. In order to identify “dose” and mechanisms associated with physical activity-induced tumor suppression, preclinical trials utilizing animal models are necessary.

Transgenic mouse models of breast cancer represent an excellent approach to dissect the mechanisms involved in exercise-induced prevention, since these models recapitulate multiple stages of breast cancer development from tumor initiation to metastasis. Hence, the effect of exercise training on any of the stages of tumor progression could hypothetically be studied. Two transgenic mouse models of mammary cancer have been reported in exercise intervention studies [4], [5], [6]. Treadmill running is the most common modality adopted for endurance exercise training in mice. Chronic (20 weeks) treadmill running reduced tumor volume in C3(1)/SV40 Tag mice relative to sedentary controls [4], with serum MCP-1 and IL-6 concentrations also lower in exercise-trained mice than in sedentary mice. Voluntary wheel running (20 weeks) was similar to long-term treadmill running in attenuating mammary tumor progression, but not tumor initiation, in C3(1)/SV40 Tag mice [5]. In contrast to the anti-tumor effects presented by these studies, 9 weeks of treadmill running exacerbated mammary tumor multiplicity and reduced survival compared with sedentary controls in the p53^+/−^-MMTV-Wnt-1 model of mammary cancer [6]. The discrepancy in the results could be due to multiple factors. For instance, the effects of exercise training may interact with p53, Wnt and C3(1)/SV40 signaling differently, which would impact the rates of tumor growth. Also, the kinetics of tumor progression are different between the p53-deficient and C3(10/SV40 cancer models. Thus, the tumor response to exercise training may already be inherently different. Finally, differences in the duration and intensity of exercise training as well as differences in murine background strains between the studies may influence tumor outcomes.

In the last decade, the scientific community has recognized the pivotal role of the tumor microenvironment in promoting tumorigenesis and tumor progression. This paradigm shift has occurred due to the appreciation that cancer is not simply a complex mass of homogenous transformed cells, but rather is comprised of a biological niche of malignant cells and other stromal cells that cross-talk and interact with one another. The complex interaction between tumors and stromal cells in the microenvironment is dynamic and influences tumor fate, either by inducing tumor cell death and dormancy or an enhancement of growth into a malignant and metastatic state. Such interactions depend on the intercellular signaling network involving various growth factors, chemokines, cytokines and other molecules secreted by stromal cells [7]. Some of the usual suspects in the stromal microenvironment include: macrophages, fibroblasts, lymphocytes, endothelial cells and adipocytes [8]. Tumor-associated macrophages (TAMs) are macrophages recruited into the tumor microenvironment and are instrumental in dictating tumor prognosis, depending on their polarization state. Macrophages activated (polarized) by Th1 cytokines associated with T-helper 1 (Th1) cells are classified as M1 macrophages, with the ability to eliminate tumor cells *in vitro* [9]. Macrophages activated by Th2 cytokines are classified as M2 macrophages and participate in driving tumor invasion and metastasis [10]. Chemokine (C-X-C motif) receptor type 4 (CXCR4), a chemokine receptor, as well as its ligand, CXCL12, are highly expressed in invasive breast cancer cells, relative to normal breast tissue, and home metastatic cells to distant lymph nodes, lungs and liver [11], [12]. CXCR4 and CXCL12 recruit monocytes [13] and regulatory T cells (Tregs) [14] into the tumor microenvironment [13]. Tregs are involved in tumorigenesis by suppressing the host immune response [15]. Specifically, Tregs suppress CD4^+^ T cell proliferation and secretion of IFNγ, while supporting the secretion of IL-10, an immune-suppressing cytokine associated with the presence of M2 macrophages [15]. Clinically, the presence of Tregs in the tumor microenvironment is positively correlated with histological tumor grade and associated with poor prognosis in patients with breast cancer [16].

In this study, we investigated whether 10 weeks of voluntary running significantly delays invasive breast cancer progression in transgenic mice and whether voluntary running modulates gene expression of cytokines and chemokines involved in tumor cross talk.

## Materials and Methods

### Transgenic mouse model of invasive breast cancer

We utilized the PyMT transgenic mouse model for this study. The PyMT oncogene is transcriptionally controlled by the mouse mammary tumor virus promoter/enhancer (MMTV). This transgenic mouse model was selected because it is an invasive and highly penetrant genetically engineered mouse model of breast cancer with stages of tumor progression that recapitulate many aspects of human breast cancer [17]. For instance, there is gradual loss of estrogen receptor (ER) and progesterone receptor (PR) gene expression. The loss of ER and PR gene expression has been reported in about 30% of human breast cancers with poor prognosis [17]. The PyMT model overexpresses the ErBb2/Neu gene, which has been reported in 20% of human breast cancers. Unlike the C3 (1) SV40 Tag model that has a 15% rate of pulmonary metastasis [18], the PyMT model demonstrates a high frequency (at least 90%) of pulmonary metastasis [19]. This makes the PyMT model attractive in terms of recapitulating the multiple stages of breast cancer progression in humans. PyMT males on a 100% FVB background were obtained from Jackson Labs and crossed with C57BL/6 females to produce F1 hybrids. Our group has experience working with this F1 hybrid, which has a reproducible rate of tumor growth and metastasis [20].

### Wheel running activity

We used voluntary wheel running as an exercise training intervention as described previously, but with modifications [21]. Running wheels were purchased from Med Associates (*Med Associates* ENV-044, Vermont, USA). Each wheel measured 15.5 cm in diameter. Running activity was monitored continuously for the entire duration of the study. A USB Interface Hub (*Med Associates* DIG-804, Vermont, USA), capable of receiving wireless signals from up to 40 wheels, was used to receive messages every 30 seconds. The running data was relayed to the Wheel Manager program (Med Associates SOF-860, Vermont, USA) for data storage before exporting to Microsoft Excel for processing. At 42 days of age (baseline), F1 hybrid females that expressed the PyMT transgene were randomized into individual housing with access to running wheels (runners, N = 13) or locked running wheels (non-runners, N = 12). Additional F1 hybrid females that did not express PyMT were randomized into the same individualized set-up with 5 mice per cohort. Throughout the experiment, mice were weighed and palpated weekly for presence of tumors in all ten mammary glands starting at 62 days of age. Once tumors were palpable, they were measured weekly with digital calipers in a blinded manner and consistently by the same individual. Tumors were measured twice in two dimensions (length x width) to obtain an estimation of surface area. All animals were housed in ventilated cages (Allentown, PA) in a specific pathogen free facility at the University of Washington. Mice were fed standard chow (5053; Picolab, Richmond, IN) and provided reverse osmosis water *ad libitum*. All supplies were autoclaved before being brought into the facility. Rooms were kept at a 12-hour light/dark cycle, maintained at 70–74°F, 45–55% humidity with 28 changes every hour. Sentinel mice were tested every quarter and were negative for standard mouse pathogens. The experimental protocol was reviewed and approved by the Institutional Animal Care and Use Committee at the University of Washington.

### Body composition

Body composition was determined at baseline, midpoint (4 weeks) and at a final time point (10 weeks). We utilized quantitative magnetic resonance (QMR; EchoMRI-100 analyzer, *Echo Medical Systems, Houston, TX*) to quantify lean and fat mass *in vivo*. In brief, mice were placed while conscious, into a plastic sample holder. This sample holder was then inserted into the QMR machine. A total of 3 measurements were obtained for each mouse and used in subsequent statistical analyses.

### Tissue harvesting

When mice reached 109–111 days of age and after 10 weeks of running, they were euthanized because this terminal endpoint has been shown to corroborate with pulmonary metastasis [20]. At necropsy, mammary tumors were weighed and measured in 3 dimensions with digital calipers. Mammary tumors from the right side per mouse were fixed in 10% formalin. Mammary tumors from the left side were flash-frozen in liquid nitrogen and stored at −80°C. Lungs were removed and individual lobes perfused with formalin. Fixed tumors and lungs were processed routinely and stained with hematoxylin and eosin (H&E). Hearts and spleens were removed and weighed.

### Histopathology

A board certified veterinary pathologist masked to the experimental groups reviewed the H&E-stained primary mammary masses and lung sections. Stained sections of primary tumors were evaluated for mitosis, necrosis and inflammation at various magnifications (20× to 400×) with a light microscope. If multiple tumor masses were present on the primary tumor slide, scores were performed on the largest tumor. Normal and aberrant mitotic figures, characterized by tri-radial or circular metaphase plates, were counted in three random fields at 400× magnification. Necrosis was estimated as the percentage of necrotic area within the largest tumor section. Inflammation was scored as 0 (minimal), 1 (mild), 2 (moderate), or 3 (marked) at 40× and 100× magnification. Invasiveness was characterized by neoplastic epithelial cells within the adjacent skeletal muscle, outside of the confines of the basement membrane. Lung sections were examined for metastatic lesions.

H&E slides were scanned in Brightfield at 20X objective using a Nanozoomer Digital Pathology slide scanner (*Olympus* America; Center Valley, PA, USA). The digital images were then imported into Visiopharm software (Hoersholm, Denmark) for analysis. Using the Visiomorph Digital Pathology module, regions of interest (ROIs) were applied around relevant areas using a tissue detect protocol and manual cleanup. Specifically, ROIs were manually drawn around tumor foci to allow differentiation of outputs between tissue in normal ROIs and in tumor ROIs. The software was then programmed to label normal tissue areas versus tumor tissue areas. Images were processed in batch using these configurations to generate the desired output calculations.

### Immunohistochemistry

We used Ki-67 as a marker for cell proliferation and F4/80 as a macrophage surface marker. Briefly, paraffin-embedded tumors were sectioned into 5 mm thick film and mounted on glass slides. Samples were processed through a series of xylene and graded alcohol washes. Antigen retrieval was carried out in commercial antigen retrieval solutions (*DAKO*, Carpinteria, CA, USA), steamed for 20 (F4/80) or 40 minutes (Ki-67) and cooled on the bench-top for 20 minutes. Primary antibodies to Ki-67 (*DAKO*), F4/80 (*Serotec*, MCA497GA, Raleigh, NC, USA) were incubated at 1∶50 for 1 hour. Sections were blocked in 15% goat/5% mouse serum. BGA rat secondary antibodies (*Jackson ImmunoResearch Labs Inc*, West Grove, PA, USA) were used and incubated at 1∶200 for 30 minutes. A streptavidin/horse-radish peroxidase tertiary reagent (SA-HRP, *ThermoScientific*, Rockford, IL, USA) was used in the Ki-67 detection. Slides were then washed twice for 4 minutes each with 3,3′-Diaminobenzidine (DAB, *DAKO*). Slides were counterstained with hematoxylin (25% hematoxylin, 75% distilled water).

Digital images were captured using a Nikon Eclipse E400 bright field microscope (*Nikon Corporation*, Tokyo, Japan) attached with a Nikon Coolpix 995, digital camera (*Nikon Corporation*, Tokyo, Japan; 3.3 megapixels, resolution 2048×1536 pixels). Five random images at 60× magnification were captured per tumor sample under blinded conditions and saved in JPG (Joint Photographic Experts Group) format. We analyzed a total of 90 digital images (with 45 images per treatment condition) for F4/80 and 90 digital images (with 45 images per treatment condition) for Ki-67 staining respectively. ImmunoRatio, an open-access image analysis software (http://jvsmicroscope.uta.fi/immunoratio/), was used to quantitate Ki-67 labeling. Positive Ki-67 staining was expressed as percentage of DAB stained nuclei relative to positive nuclei. ImageJ (*Image J*, version 1.44o, developed by Wayne Rasband, National Institutes of Health, Bethesda, Maryland, USA) was utilized to quantitate F4/80 staining by employing color thresholding. In brief, digital images were opened in ImageJ, duplicated and segmented into three color channels (red, green, blue). The blue channel was selected for quantification of positive F4/80 staining. A threshold was selected for the area stained positive for F4/80 and the resulting area considered the area of positive stain. The red channel was selected for quantification of overall nuclear staining, and a threshold was also applied. The positive F4/80 area to nuclear area was reported as percentage of DAB immunostain relative to total nuclei area.

### Gene expression

We were interested in the effects of voluntary running on the gene expression of the macrophage markers CD163 and CD206 for the M2 phenotype and CXCL10 for the M1 phenotype, the tumor invasion and metastasis markers CXCR4, CXCL12, and the T-regulatory cell marker CCL22. Frozen mammary tumors were placed in autoclaved Eppendorf tubes pre-filled with a commercial tissue lysis buffer (Buffer RLT, *Qiagen*, Valencia, CA, USA). Sample tubes were then placed in a tissue homogenizer (Bullet Blender, *Next Advance*, NY) for homogenization. Total cellular RNA was isolated from mammary tumor homogenates using a commercial RNA isolation kit (RNeasy, *Qiagen*). RNA purity was first determined with a Nanodrop spectrophotometer (*ThermoScientific*) and the A260/280 and A260/230 ratios were greater than 1.8 for all samples. RNA quality was further validated electrophoretically by inspecting 18S and 28S band intensities using an Agilent 2100 Bioanalyzer (*Agilent*, Santa Clara, CA, USA).

Reverse transcription was completed using SuperScript III First-Strand Synthesis SuperMix for qRT-PCR (*Invitrogen Inc*., Carlsbad, CA, USA) following the manufacturer's established protocol with 1 ug total RNA starting material. Quantitative RT-PCR was performed on ABI PRISM 7900HT System (*Applied Biosystems Inc*., Foster City, CA, USA) using a fluorogenic 5′ nuclease-based FAST assay 12 ul reaction containing 20 ng cDNA, 0.5X TaqMan Gene Expression Fast Universal Master Mix (Applied Biosystems, Inc.), and 0.4X Inventoried Assay Mix (Applied Biosystems Inc.). The reaction consisted of denaturing at 95°C for 15 s followed by 40 cycles of 95°C for 1 s and 60°C for 20 s. Data analysis was completed using Sequence Detection Systems V2.2.2 (*Applied Biosystems Inc*). 18S amplification plots derived from serial dilutions of an established reference sample were used to create a linear regression formula to calculate expression levels, and 18S was used as an internal control to normalize the data.

For CXCR4, reverse transcription was carried out using Quantitect reverse transcription kit (*Qiagen*) according to the manufacter's established protocol. A total of 40 ng of RNA was reverse transcribed to cDNA. Quantitative RT-PCR was performed on the Rotor Gene 3000 real time cycler (*Qiagen*), using SYBR green mastermix (*Qiagen*). The reaction consisted of denaturing at 95°C for 10 minutes, followed by 40 cycles of 95°C for 7 s and 60°C for 15 s. Data analysis was performed using the standard curve method and normalized to 18S. Primer sequences for CXCR4 were obtained from Primer bank (Wang & Seed, 2003).

### Statistical analyses

Student's t-test was used to detect significant differences between groups for the following dependent variables: tumor area, tumor burden, cardiac weight, body weight and fat mass. Pearson's correlation was used to determine the relationship between running distance and tumor sizes, as well as between tumor sizes and anthropometric measures (body mass, fat mass, lean mass). Changes in body fat mass, running distance and body weights were assessed using a repeated measures analysis of variance (ANOVA) with Bonferroni post-test analysis. The data for spleen weights were assessed using a 1-way ANOVA with Tukey's post-test analysis. Prism (Graph Pad) and SigmaPlot (Version 11.0, Systat Software, Inc.) were used to perform the statistical analyses. All results are presented as means ± standard deviation.

## Results

### Running attenuates early tumor growth

**Figure 1:**
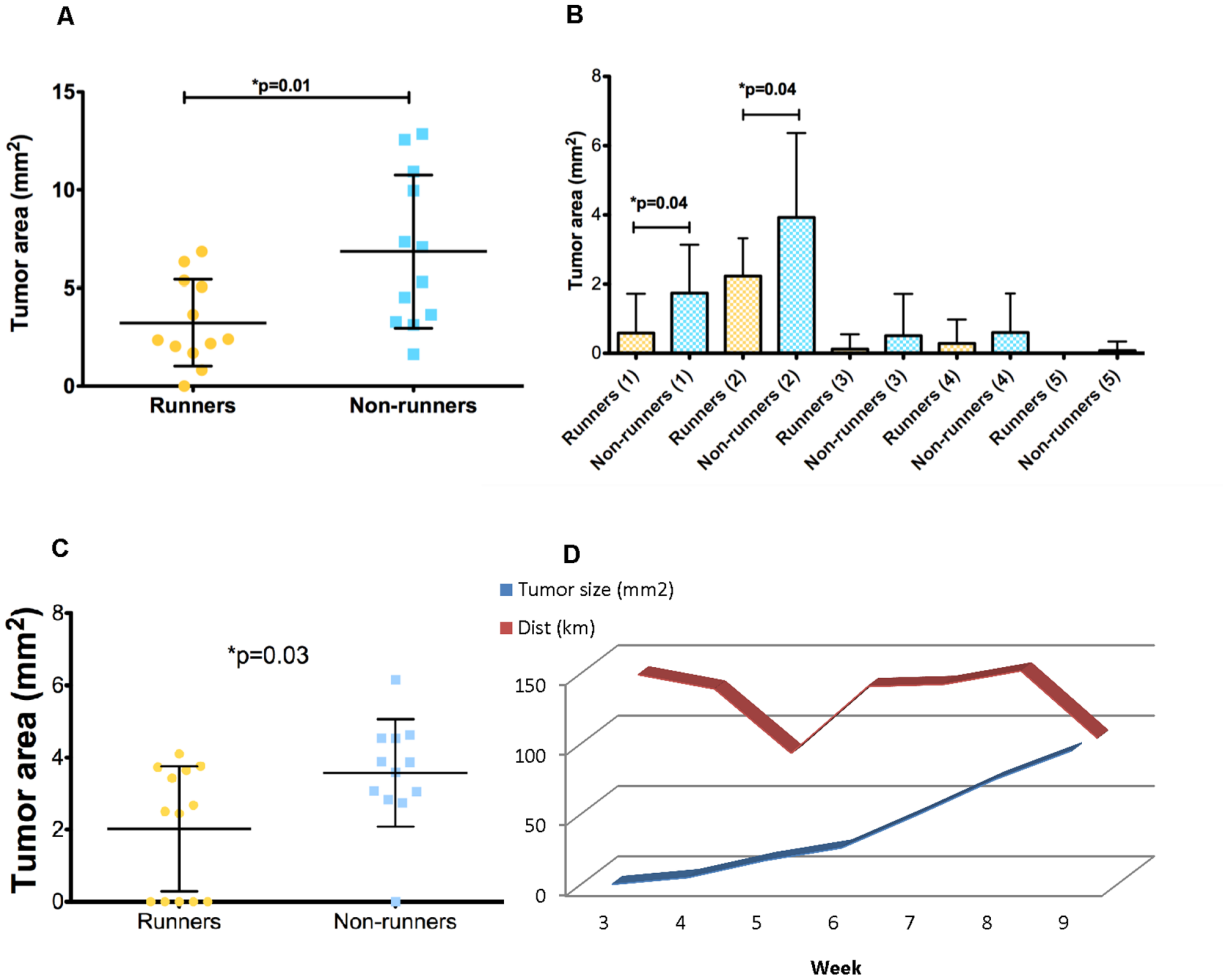
The surface area of breast tumors in PyMT transgenic mice was decreased after three weeks of voluntary wheel running. Values are means ± standard deviation (N = 12 per group). A. Size of all breast tumors in runners was significantly decreased compared to non-runners (p = 0.01). B. Differences in tumor sizes were observed across the anterior (1–3) and posterior (4–5) mammary glands (p = 0.04). C. Runners have a significant decrease in tumor size in mammary gland 1 after 5 weeks of running (p = 0.03). D. Runners showed a biphasic pattern in the weekly distance ran (km). Tumor growth showed a weekly increase.

Significant differences were detected in sizes of palpable tumors from runners, showing a reduced surface area after three weeks of running, compared with non-runners (p = 0.01) (Figure 1A). The largest tumors in both runners and non-runners were in mammary glands 1 and 2, which by themselves demonstrated a significant decrease in tumor size with running (p = 0.04) (Figure 1B). This significant effect of voluntary wheel running on overall tumor progression was no longer evident after 4 weeks of running, although the tumor sizes in the first pair of mammary glands were significantly smaller in runners compared with non-runners up until the fifth week (p = 0.03) (Figure 1C). Runners showed a biphasic pattern in the weekly distance ran, while tumor growth showed a weekly increase (Figure 1D).

### Decreased tumor growth is associated with increased running distance

**Figure 2:**
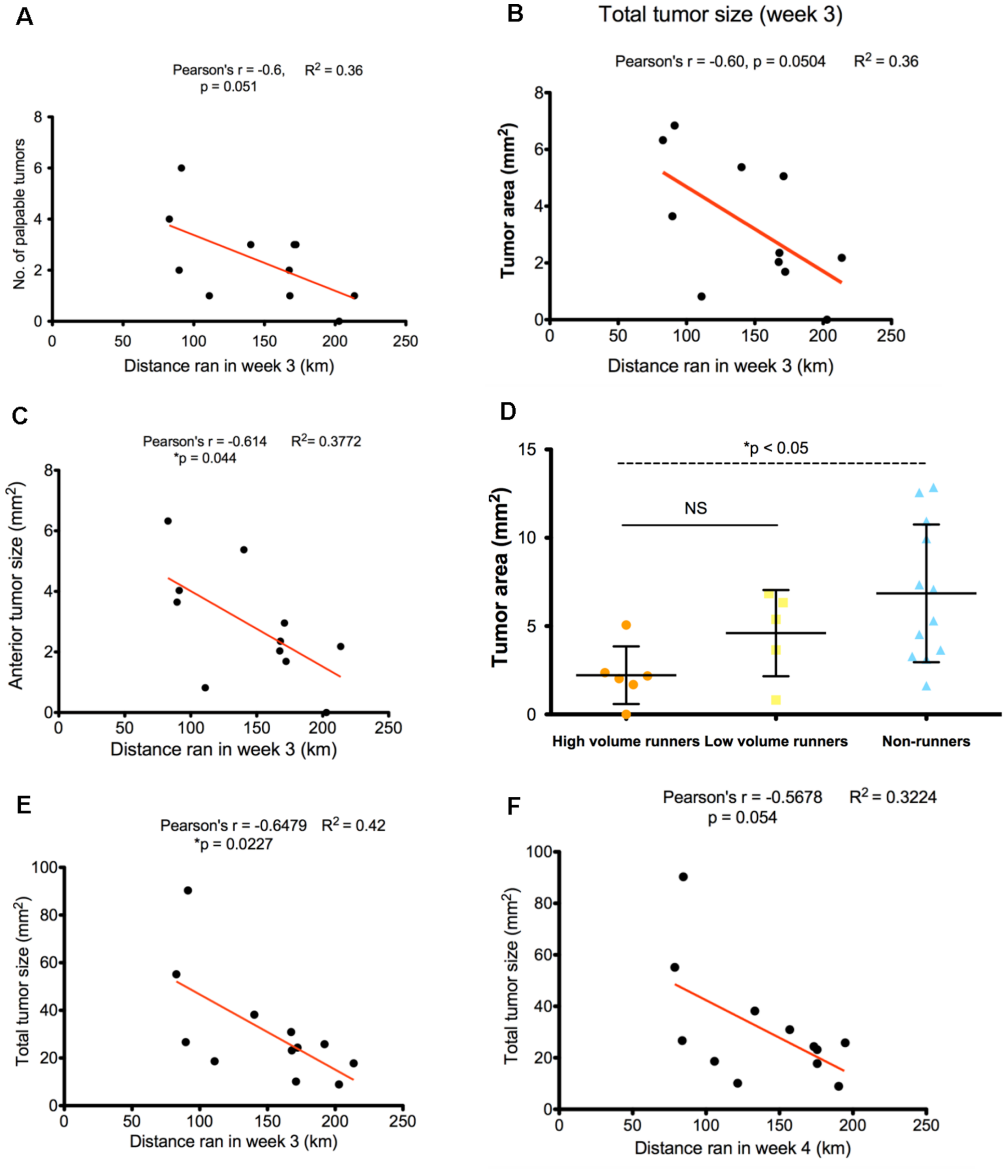
The number and size of breast tumors in PyMT mice correlated with distance run. **A**. Distance run at 3 weeks was associated with the number of palpable tumors during the 3rd week (Pearson's r = −0.6, p = 0.051, R^2^ = 0.36), **B**, total tumor sizes (Pearson's r = −0.6, p = 0.0504, R^2^ = 0.36), and **C**, anterior tumor sizes (glands 1–3) (Pearson's r = − 0.61, p = 0.044, R^2^ = 0.38). **D**. High volume runners demonstrated significantly smaller tumor sizes compared with non-runners during the 3rd week (p<0.05), but not with low volume runners. **E**. Distance ran at 3 weeks was correlated with total tumor size in runners during the 5th week (Pearson's r = −0.65, p = 0.023, R^2^ = 0.42). **F**. Distance run at 4 weeks was correlated with total tumor size in runners during the 5th week (Pearson's r = −0.57, p = 0.054, R^2^ = 0.32).

We found a dose-dependent correlation between the distance run during the third week and tumor growth. A correlation was observed for distance ran after 3 weeks on the number of palpable tumors (Pearson's r = −0.6, p = 0.05, R^2^ = 0.36) (Figure 2A) and total tumor size, (Pearson's r = −0.6, p = 0.05, R^2^ = 0.36) in the 3rd week (Figure 2B). This relationship was observed for distance ran after 3 weeks on anterior tumor sizes (Pearson's r = −0.61, p = 0.04, R^2^ = 0.38) (Figure 2C), which correspond to tumors located in mammary glands 1 and 2. After stratifying runners into long distance (>150 km/week) or short distance (≤150 km/week), we observed a significant difference in total tumor size between long distance runners and non-runners (p≤0.05) (Figure 2D) but not between long distance runners and short distance runners, nor with short distance runners and non-runners. The distance ran during the third and fourth weeks was inversely correlated with total tumor size during week 5, respectively (Pearson's r = −0.65, p = 0.02, R^2^ = 0.42; Pearson's r = −0.57, p = 0.05, R^2^ = 0.32) (Figures 2E and 2F).

### Runners with decreased tumor burden have increased fat mass

**Figure 3:**
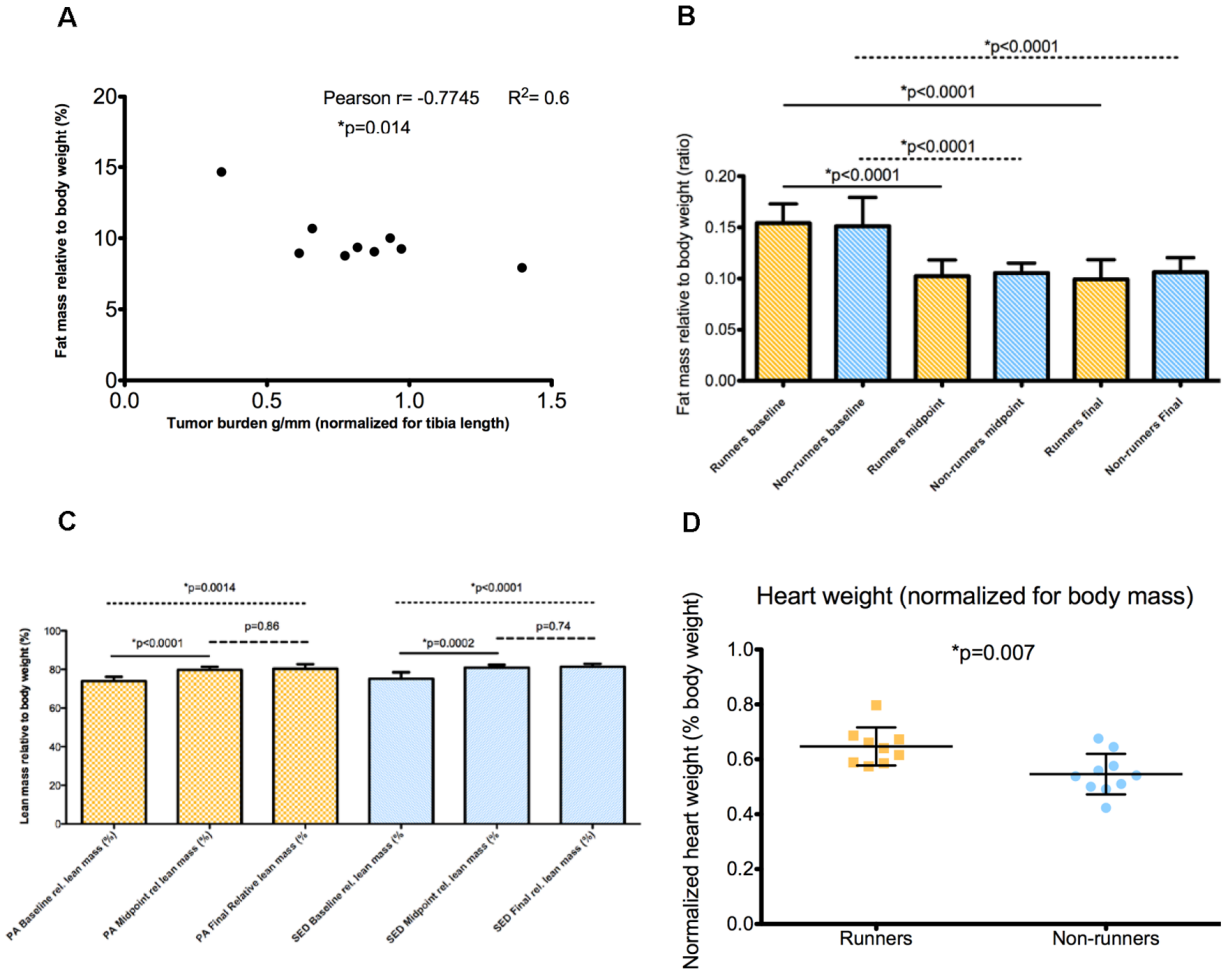
Runners with increased fat mass had decreased tumor burden. **A**. The amount of adiposity at sacrifice was significantly correlated with tumor burden in runners (Pearson's r = −0.77, p = 0.01, R^2^ = 0.6). **B**. Both runners and non-runners showed a time-dependent fat loss, and **C**. increased lean mass. **D**. Runners had increased heart weights compared to non-runners.

A correlation between increased adiposity and decreased tumor burden at the end of the study was observed in runners (Pearson's r = −0.77, p = 0.01) (Figure 3A). This suggests that increased adiposity in runners might reflect decreased adipose tissue cachexia, a common co-morbidity in breast cancer. Both runners and non-runners demonstrated significant fat loss at midpoint and at the end of the study, compared with baseline (p≤0.0001) (Figure 3B). There was an increase of relative lean mass in both runners and non-runners between baseline and at the end of the study (Figure 3C). Mice in the running group showed an increase in heart mass (normalized to body mass) compared with non-runners (p = 0.007) (Figure 3D).

### Non-runners with cancer had increased spleen weights compared to runners with no cancer

**Figure 4:**
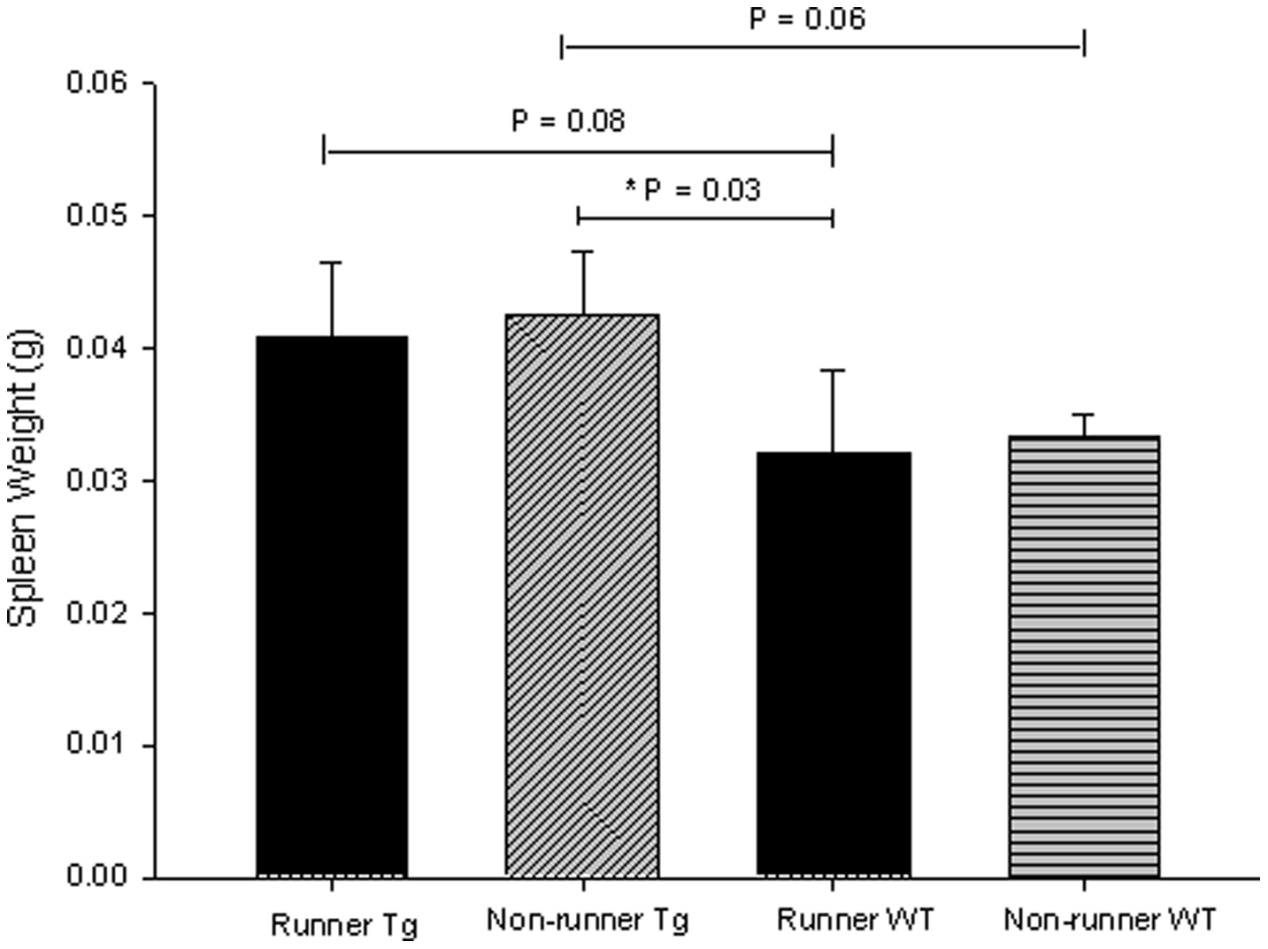
Spleen weights obtained at sacrifice in transgenic (N = 7 runners and 8 non-runners) and wild-type mice (N = 3 per condition) were increased in transgenic non-runners compared with wild-type runners (p = 0.03), but no other significant differences were observed.

Spleeen weight has been used as a surrogate biomarker for immune function and inflammation in transgenic mouse models of breast cancer [4], [5]. In this present study, we observed a significant difference in spleen weights across transgenic runners, non-runners and wild-type runners and non-runners, p = 0.01, *F* = 4.945 (Figure 4). There were no significant differences in spleen weight (normalized for body weight) at sacrifice between transgenic runners and non-runners, or between wild-type (F1 hybrids not expressing the PyMT transgene) runners and non-runners. Compared between genotypes, transgenic runners demonstrated higher spleen weights than wild-type runners, but this did not reach statistical significance. Spleen weights from transgenic and wild-type non-runners demonstrated the same pattern, while wild type runners had lower spleen weights, compared with transgenic non-runners (p = 0.03).

### Running alters cytokine gene expression in primary tumors

**Figure 5:**
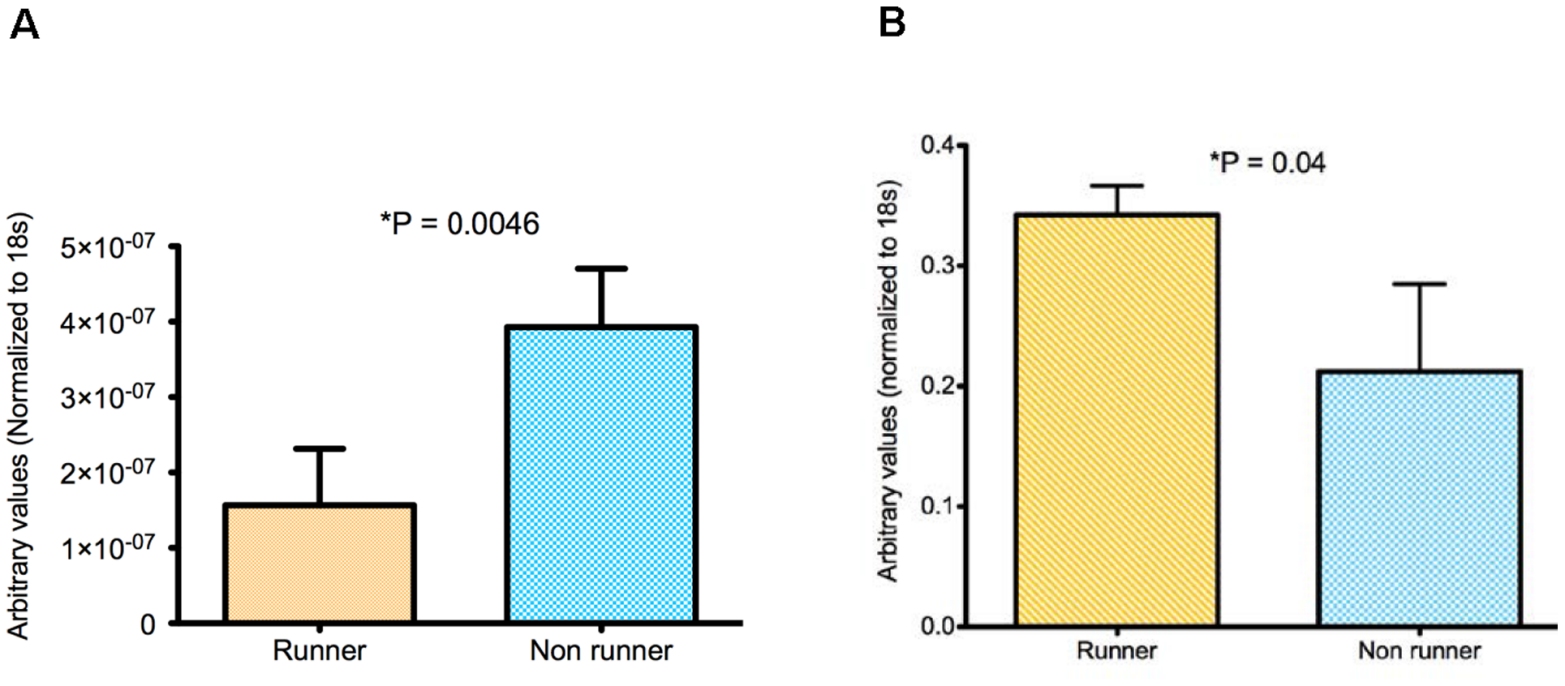
Gene expression of CCL22 but not CXCR4 is attenuated in tumors from runners but not non-runners. **A**. Tumors from runners showed four fold decrease in CCL22 (p = 0.0046, N = 4 per condition). **B**. Tumors from runners had increased expression of CXCR4 (p = 0.04, N = 3 per condition).

A significant attenuation in CCL22 gene expression was detected in primary tumors from runners (p = 0.005) (Figure 5A). Since macrophages are associated with increased CCL22 gene expression [22], we tested for gene expression of CD163 and CD206 (representative of M2), and CXCL10 (representative of M1), and found no differences. We also showed no differences in macrophage density in tumors by immunohistochemistry for F4/80, a marker of mouse macrophages. These preliminary observations suggest that voluntary wheel running most likely does not affect recruitment or polarization of TAMs. A decrease in CCL22 would suggest a decreased population of Tregs in tumors of mice that ran, since increased CCL22 gene expression is linked with increased recruitment of immunosuppressive Tregs [23].

We found a 1.5-fold increase in gene expression of the metastatic marker CXCR4 in breast tumors from runners compared with non-runners (p = 0.04) (Figure 5B). These results were surprising, as morphometric analyses of pulmonary metastatic foci and the proliferative index, as indicated by comparable levels of labeling for Ki-67, showed no differences between runners and non-runners (data not shown). There was no difference in gene expression of CXCL12, the ligand for CXCR4 [24] associated with tumor invasion and metastasis [11].

## Discussion

Our study demonstrates an early effect of voluntary wheel running on attenuating breast cancer progression in PyMT transgenic mice. We found a significant correlation between the distance ran by the mice and tumor sizes at week 3. Mice running the furthest in week 3 presented with the smallest tumor sizes, and the tumor responsiveness was influenced by the mammary gland location, in this case, anterior (glands 1 and 2). When stratifying mice into long and short distance runners, we found that long distance runners showed significantly reduced tumor sizes compared with non-runners. The distance ran at week 3 was also inversely correlated with total tumor sizes and gland 1 tumor sizes at week 5. There appeared to be a biphasic response in distance covered by runners during the course of the study, with an increase in distance from week 1 to week 3, a decrease in week 5 and a subsequent increase again in weeks 6 through 8. This biphasic response appeared to be related to tumor growth, in that early tumor growth was inversely related to total weekly distance at week 3, and positively related to total weekly distance at week 8. We suggest that 3 to 5 weeks of running attenuates tumor growth in the PyMT mouse model, with the degree of attenuation dependent on the distance ran, illustrating a dose-response relationship. We speculate that after 5 weeks of running, any additive running is no longer effective in this model. The biphasic nature of the running distance pattern argues against the increasing tumor volumes having any effect on distance ran on a week by week basis (see Figure 1D).

Both runners and non-runners experienced fat loss during the study suggesting the presence of tissue cachexia. Fat loss between the two groups was not different at midpoint, or at the final time point, suggesting that physical activity did not mitigate this loss of adiposity, rather, that it was an effect of tumor progression. Adipose tissue cachexia is a common co-morbidity experienced by breast cancer survivors [25]. Tumor growth necessitates increased substrate utilization and the adipose depot of lipids is a rich source of fatty acids for tumor metabolism. We did observe an inverse correlation between tumor burden and adiposity in runners but not in non-runners. We speculate that this inverse correlation may be due to the variable response of the mice to the training effect of voluntary wheel running or to the amount of voluntary running that was undertaken by the mice, which was not uniform. However, we did not observe any trend between distance ran and adiposity in runners, suggesting that this inverse relationship between tumor burden and adiposity may be partly mediated by other mechanisms. There was an increase of relative lean mass in both runners and non-runners between baseline and at the end of the study. The increased lean mass was probably due to developmental growth and unlikely caused by voluntary running, as both groups of mice experienced the same increase, with no difference between groups. This is consistent with a study by Allen et al. [26], where mice given access to running wheels showed no differences in their skeletal muscle weights compared with either sedentary controls or with baseline controls. We detected significant augmentation of cardiac mass in runners compared with non-runners, suggesting a training adaptation to wheel running.

CCL22 is a chemokine produced by B cells, myeloid dendritic cells, macrophages and epithelial cells in the tumor microenvironment [27]. Secreted CCL22 binds to the C-C chemokine receptor 4 (CCR4) on circulating Tregs, that migrate into the tumor microenvironment [27]. M2 macrophages have increased CCL22 gene expression [22], [28] and secrete this chemokine to recruit immunosuppressive Tregs [23]. We observed a significant decrease in CCL22 mRNA expression in tumor tissue from runners, suggesting that decreased signaling by M2 macrophages or other stromal cells in the tumor microenvironment may result in a reduction in Treg recruitment. Our results are compatible with a recent study [29] that showed decreased percentages of splenic CD4^+^CD25^+^ Tregs in tumor-bearing BALB/c mice after 8 weeks of physical activity. Similar to our study, this study did not demonstrate whether tumor bearing mice had reduced recruitment of Tregs. To determine whether exercise training directly attenuates CCL2-mediated Treg recruitment, it would be necessary for follow-up studies to identify and quantitate Tregs in breast tumors. Healthy women participating in regular exercise had significantly lower percentage of circulating CD4^+^/CD25^+^/FOXP3 Tregs, compared with women that were more sedentary [30].Thus, exercise training during cancer treatment may help reduce the immunosuppressive effects of Tregs. Since CCL22 inhibits T cell cytotoxicity, exercise training concurrently with T cell-targeted immunotherapy has clinical implications.

We did not detect significant differences in gene expression of CD163 and CD206 representing M2 TAMs or CXCL10 representing M1 TAMs in tumors from either runners or non-runners. Mice in this study were sacrificed when the tumors were fulminant and metastatic, and we have already shown that running had no anti-tumor effect at this late stage of the disease. No significant differences were observed in immuno-histochemical expression of F4/80 in tumor tissue between runners and non-runners. Therefore, running was not able to alter macrophage recruitment or TAM polarization at a time when mice were affected by an overwhelming disease burden. Expression of M1 markers is more likely to be expressed earlier, during the initiation phase [31]. Since no mice in our study were terminated early after only three or four weeks of running, we did not have tumor tissue to analyze for TAM phenotypes so do not know if running had any effect on TAM polarization at a time when runners had decreased tumor burden. TAMs have been associated with metastasis in the PyMT model of breast cancer, [17], but we saw no differences in metastasis in runners and non-runners, so it is possible that TAMs may not be an anti-tumor target for exercise training activities as previously suggested [10]. We noted an increase in the expression of the metastatic marker CXCR4 in tumor tissue from runners compared to non-runners, suggesting no correlation with TAMs. It is possible that the increase in CXCR4 gene expression may be due to the recruitment of other immune cells and hematopoietic progenitor cells into the tumor microenvironment [32], but the biological significance in this context remains unknown.

Pre-clinical animal models provide a useful platform to assess quantifiable measures of exercise training on tumor outcomes. Different animal models of breast cancer have been utilized in exercise and physical activity intervention studies. Carcinogen-induced models commonly involve injecting animals with dimethylbenz (a) anthracene (DMBA) [33], [34] or 1-methyl-1 nitrosurea (MNU) [35]. One limitation with using a carcinogen-induced mammary cancer model is that any main effect of exercise training on tumor progression may be confounded by its effect on carcinogen detoxification [36], and not on the tumor *per se*. Animals are susceptible at higher doses of carcinogen exposure, whereas humans succumb at much lower doses [36]. Subcutaneous injection of tumor cells is another common method for inducing cancer in rodents. This method has limitations for translation, because the injection site is not organ-specific. Orthotopic mammary cancer models, such as the 4T1 syngeneic BALB/c model [37], while being tumor-site specific, have thus far not been examined in the context of exercise or physical activity. Other groups have studied the effects of exercise using human xenografts in orthotopic models but within the context of an immunosuppressed host, such as in athymic mice [38], [39]. To the best of our knowledge, there are only a limited number of studies that have investigated the anti-tumor effects of exercise training or physical activity in transgenic mouse models of breast cancer. Our results corroborate those of Murphy *et al*. [4] and Steiner *et al*. [5], demonstrating that exercise training attenuates tumor progression. The difference between our studies is that we noted an earlier effect of exercise training on mitigating tumor progression, whereas they found a significant effect towards the later phases. This demonstrates that different transgenic models respond differently to exercise, likely as a consequence of gene-exercise interactions, thus making a direct comparison between models challenging.

In conclusion, we report that after three weeks of voluntary wheel running, PyMT transgenic mice have decreased tumor sizes, with mice running the furthest distance showing the smallest tumors. This suggests a dose-dependent relationship between exercise and the suppression of tumor growth. This positive affect disappears over the next several weeks of running as the tumors continue to grow and overwhelm the system, but with an indication that cytokine signaling for immunosuppressive regulatory T cells may be down regulated by exercise. These observations suggest that the initiation of moderate exercise training for adjunctive therapeutic benefit early in the course of invasive breast cancer should be considered for further investigation.
